# A maternal high-fat diet during pregnancy and lactation, in addition to a postnatal high-fat diet, leads to metabolic syndrome with spatial learning and memory deficits: beneficial effects of resveratrol

**DOI:** 10.18632/oncotarget.22960

**Published:** 2017-12-06

**Authors:** Shih-Wen Li, Hong-Ren Yu, Jiunn-Ming Sheen, Mao-Meng Tiao, You-Lin Tain, I-Chun Lin, Yu-Ju Lin, Kow-Aung Chang, Ching-Chou Tsai, Li-Tung Huang

**Affiliations:** ^1^ Department of Pediatrics, Kaohsiung Chang Gung Memorial Hospital and Chang Gung University College of Medicine, Kaohsiung, Taiwan; ^2^ Department of Medical Research, Kaohsiung Chang Gung Memorial Hospital, Kaohsiung, Taiwan; ^3^ Department of Obstetrics and Gynecology, Kaohsiung Chang Gung Memorial Hospital and Chang Gung University, College of Medicine, Kaohsiung, Taiwan; ^4^ Anesthesiology, Kaohsiung Chang Gung Memorial Hospital and Chang Gung University College of Medicine, Kaohsiung, Taiwan; ^5^ Department of Obstetrics and Gynecology, Kaohsiung Chang Gung Memorial Hospital and Chang Gung University, College of Medicine, Kaohsiung, Taiwan; ^6^ Department of Traditional Medicine, Chang Gung University, Linkow, Taiwan

**Keywords:** maternal high-fat diet/obesity, spatial, resveratrol, postnatal high-fat diet, hippocampus

## Abstract

We tested the hypothesis that high-fat diet consumption during pregnancy, lactation, and/or post weaning, altered the expression of molecular mediators involved in hippocampal synaptic efficacy and impaired spatial learning and memory in adulthood. The beneficial effect of resveratrol was assessed. Dams were fed a rat chow diet or a high-fat diet before mating, during pregnancy, and throughout lactation. Offspring were weaned onto either a rat chow or a high-fat diet. Four experimental groups were generated, namely CC, HC, CH, and HH (maternal chow diet or high-fat diet; postnatal chow diet or high-fat diet). A fifth group fed with HH plus resveratrol (HHR) was generated. Morris water maze test was used to evaluate spatial learning and memory. Blood pressure and IPGTT was measured to assess insulin resistance. Dorsal hippocampal expression of certain biochemical molecules, including sirtuin 1, ERK, PPARγ, adiponectin, and BDNF were measured. Rats in HH group showed impaired spatial memory, which was partly restored by the administration of resveratrol. Rats in HH group also showed impaired glucose tolerance and increased blood pressure, all of which was rescued by resveratrol administration. Additionally, SIRT1, phospho-ERK1/2, and phospho-PPARγ, adiponectin and BDNF were all dysregulated in rats placed in HH group; administration of resveratrol restored the expression and regulation of these molecules. Overall, our results suggest that maternal high-fat diet during pregnancy and/or lactation sensitizes the offspring to the adverse effects of a subsequent high-fat diet on hippocampal function; however, administration of resveratrol is demonstrated to be beneficial in rescuing these effects.

## INTRODUCTION

Maternal obesity is a pervasive health issue, with over 30% of child-bearing age women being categorized as obese [1]; the trend of obesity has continually increased between 2005 and 2014 [2]. In addition, maternal high-fat diet/obesity may predispose the offspring to altered energy balance, cardiovascular dysfunction, neuroinflammation, and obesity [3–7]. Furthermore, a combined maternal high-fat diet and postnatal high-fat diet increases the risk of metabolic syndrome in the offspring [8–10].

Metabolic syndrome refers to a cluster of risk factors including obesity, dyslipidemia, hypertension, and insulin resistance [11]. Metabolic syndrome is known to affect cognition and contributes to the development and progression of Alzheimer’s disease [12, 13]. We have previously reported that a combined maternal high-fat diet and a postnatal high-fat diet resulted in obesity and hypertension [14]. However, it remains unclear whether a maternal high-fat diet/obesity could program offspring to develop metabolic syndrome upon exposure to a high-fat diet post-weaning. Additionally, the effect of this dual exposure to a high-fat diet on the development of cognitive deficits is yet to be determined.

Resveratrol, a natural polyphenolic compound, is produced by plants in response to environmental stress and is found in red grape skin, peanuts, a variety of berries and medicinal plants. Resveratrol possesses important biological properties, including antioxidant, anti-inflammation and neuroprotective effects [15, 16]. Resveratrol is also able to enhance hippocampal plasticity and hippocampal neurogenesis [17]. Recently, a number of studies have focused on the neuroprotective effects of resveratrol, including its ability to diminish the toxicity induced by the amyloid beta peptide [18, 19] and kainic acid [20], as well as prevent cerebral ischemic damage [16, 21]. In addition, animal studies have demonstrated the efficacy of resveratrol to reverse learning and memory impairments in aged animals [16, 22].

Thus, the purpose of this study was to investigate the effects of maternal high-fat diet during pregnancy and lactation alone, as well as in combination with a postnatal high-fat diet, on metabolic profiles and spatial learning and memory in adult male offspring. Hippocampal SIRT1, adiponectin, ERK1/2, phospho-insulin receptor substrate-1 (phospho-IRS-1), tumor necrosis factor-α (TNF-α), p66Shc and BDNF levels were examined. In addition, the effect of resveratrol was assessed.

## RESULTS

### Body weights

**Figure 1:**
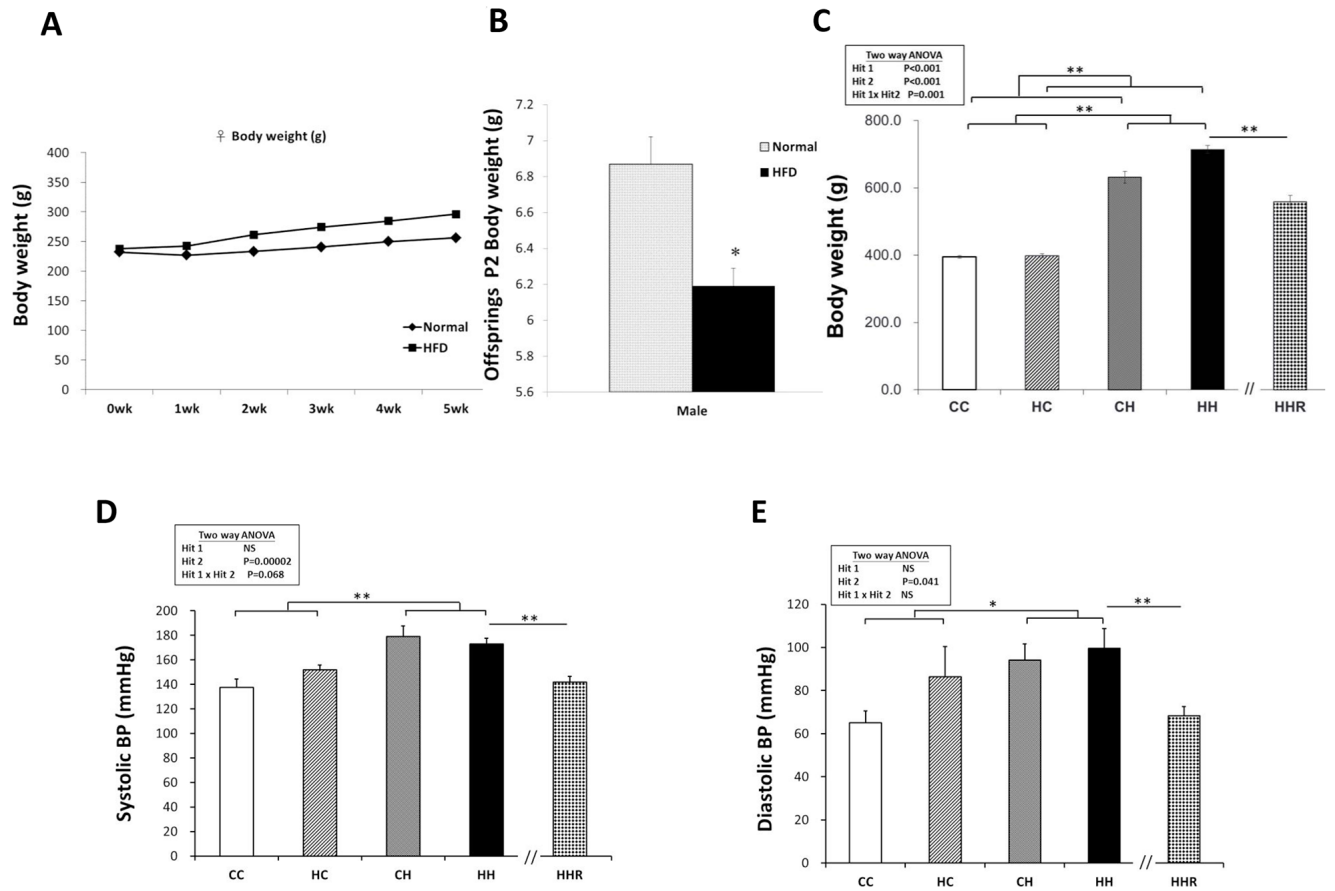
Body weight and blood pressures **(A)** Female rats were fed a chow diet or a high-fat diet and weighed weekly for 5 weeks starting from conception. **(B)** After mating, male pups were weighed on PND 2. The offspring were weaned at 3 weeks of age, and assigned to either the chow diet or high-fat diet ad libitum from weaning up to 4 months of age. **(C)** The male rats were weighed at 4 months. Body weights were analyzed using two-way ANOVA (maternal diet x post-weaning diet) and the therapeutic effect of resveratrol was evaluated by Student *t*-tests. Hit 1 indicated CC and CH groups vs. HC and HH groups. Hit 2 indicated CC and HC groups vs. CH and HH groups. **(D)** Systolic and **(E)** diastolic blood pressures of 4-month old male rats were analyzed using two-way ANOVA and the therapeutic effect of resveratrol was evaluated by Student *t*-tests. ^*^ P<0.05; ^**^ P<0.01.

Before mating female rats were fed a chow diet or a high-fat diet and weighed weekly starting from conception until 5 weeks post conception (Figure 1A). High-fat diet dams had significantly heavier body weight on gestational day 19 compared to chow fed dams (353±10.44 gm vs. 413.68±10.86 gm; P<0.01). Female rats were separated into a control group and an obesity group. After birth, male offspring were weighed on PND 2 (Figure 1B). The offspring were weaned at 3 weeks of age, and assigned to either the chow diet group or the high-fat diet group; Food was provided *ad libitum* from weaning to 4 months of age. The male rats were weighed again when they were 4 months old (Figure 1C). Our results show that the male offspring from dams with maternal obesity had decreased birth weight. Analysis of weight taken when offspring were 4 months old, shows a significant main effect of both maternal obesity/high-fat diet [F(1,76)=13.449, P<0.001], and a postnatal high-fat diet treatment [F(1,76)=554.604, P<0.001]. There was also a significant interaction effect between maternal obesity/high-fat diet and postnatal high-fat diet [F(1,76)=11.757, P=0.001]. Adult offspring in the HH group were the heaviest, followed by members of the CH group. Analysis using the Student’s *t*-test showed a significant effect of resveratrol treatment to decrease body weights in animals exposed to both treatments (HH vs. HHR; P<0.001).

### Biochemistry parameters of dams

On day 19 of lactation, the biochemistry parameters showed increased levels of AST, ALT, cholesterol, triglyceride and plasma glucose in high-fat diet/obesity rats (p<0.05), whereas HDL was decreased in high-fat diet/obesity rats (p<0.05) (Table 1). Together with increased gestational body weight, these data indicated that a high-fat diet caused obesity in pregnant dams.

**Table 1:** Biochemistry parameter of lactating dams

	Dams	
	Chow diet N=7	High-fat diet/obesity N=12
**AST (U/L)**	83.6±4.08	143.0±12.72^**^
**ALT (U/L)**	37.3±5.53	97.8±8.3^**^
**Cholesterol (mg/dL)**	63.0±3.83	96.0±8.23^**^
**Triglyceride (mg/dL)**	45.2±7.26	75.9±8.97^*^
**HDL (mg/dL)**	52.7±3.53	44.5±3.17^*^
**Glucose (mg/dL)**	214.5±7.08	256.1±12.33^*^

Values are means ± SEM; AST, aspartate aminotransferase; ALT, alanine aminotransferase; HDL, high-density lipoprotein. Data were collected from dams on lactating day 19. ^*^ P< 0.05, ^**^ P<0.01.

### Blood pressure

The blood pressure of 4-month-old male rats was measured (Figure 1D and 1E). We observed a significant main effect for the postnatal high-fat diet treatment [F(1,14)=24.514, P<0.001], however, there was no significant effect of maternal obesity/high-fat diet on systolic blood pressure. A two-way ANOVA showed no significant interaction effects between maternal obesity/high-fat diet and a postnatal high-fat diet. This result indicated that a postnatal high-fat diet caused increased systolic blood pressure. Additionally, a Student’s *t*-test analysis showed that resveratrol treatment significantly restored systolic blood pressure levels in these animals (HHR vs. HH; P=0.006). Similarly, we observed a main effect for the postnatal high-fat diet treatment [F(1,14)=5.046, P=0.041], however, maternal obesity/high-fat diet did not significantly affect diastolic blood pressure. A two-way ANOVA showed no significant interaction between maternal obesity/high-fat diet and postnatal high-fat diet. Further analysis using a Student’s *t*-test showed that resveratrol treatment significantly restored diastolic blood pressure (HHR vs. HH; P=0.009).

### Intraperitoneal glucose tolerance test (IPGTT)

**Figure 2:**
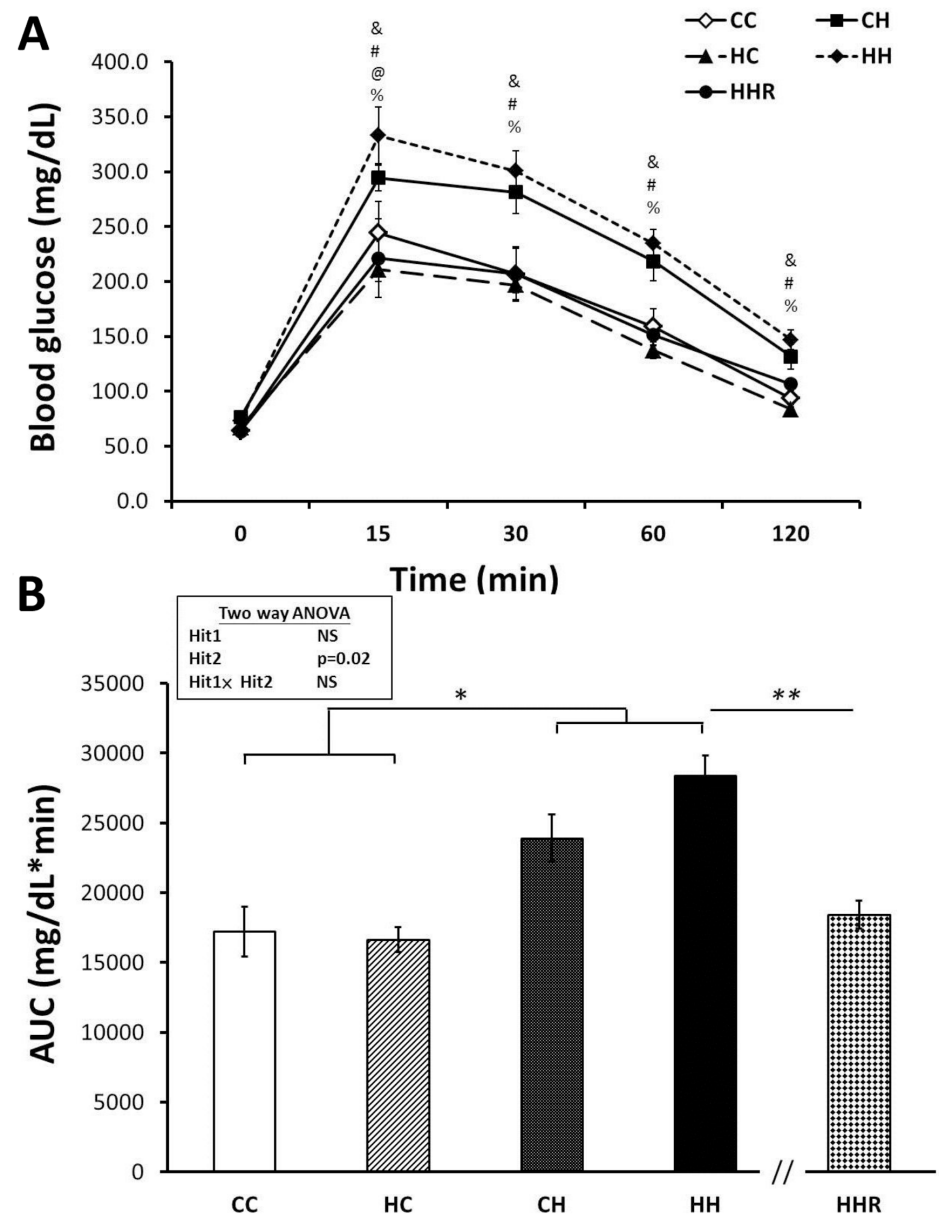
Blood glucose levels of post-stimulation tests **(A)** Intraperitoneal glucose tolerance test. **(B)** Glucose area under the curve (AUC). Results were analyzed using two-way ANOVA (maternal diet x post-weaning diet). ^&^: P<0.05, HH versus CC; ^#^: P<0.05, HH versus HC; ^@^: P<0.05, HH versus CH; ^%^: P<0.05, HH versus HHR; ^*^ P<0.05; ^**^ P<0.01.

The blood glucose levels of rats in the CH and HH groups were measured at the 15, 30, 60 and 120 min mark, Results indicate that blood glucose levels of rats in the CH and HH groups were higher compared with those of the CC group (Figure 2A). However, rats treated with resveratrol had blood glucose levels similar to those of rats in the CC group (Figure 2A). A two-way ANOVA of AUC showed no significant main effect of maternal obesity/high-fat diet treatment [F(1,52)=0.03, P=0.862], however, we observed a significant main effect of postnatal high-fat diet treatment [F(1,52)=5.76, P=0.002]. There was no interaction effect of maternal obesity/high-fat diet and postnatal high-fat diet in AUC [F(1,52)=3.039, P=0.087]. A Student’s *t*-test showed that resveratrol treatment significantly decreases the AUC (HH vs. HHR; P<0.01). These results indicate that the AUC was larger in the CH and HH groups compared to the CC and HC groups (Figure 2B), and treatment with resveratrol restored the AUC to levels similar to that observed in controls. Thus, the data shows that exposure to a postnatal high-fat diet results in peripheral insulin resistance and resveratrol treatment is able to reverse this effect.

### Morris water maze

**Figure 3:**
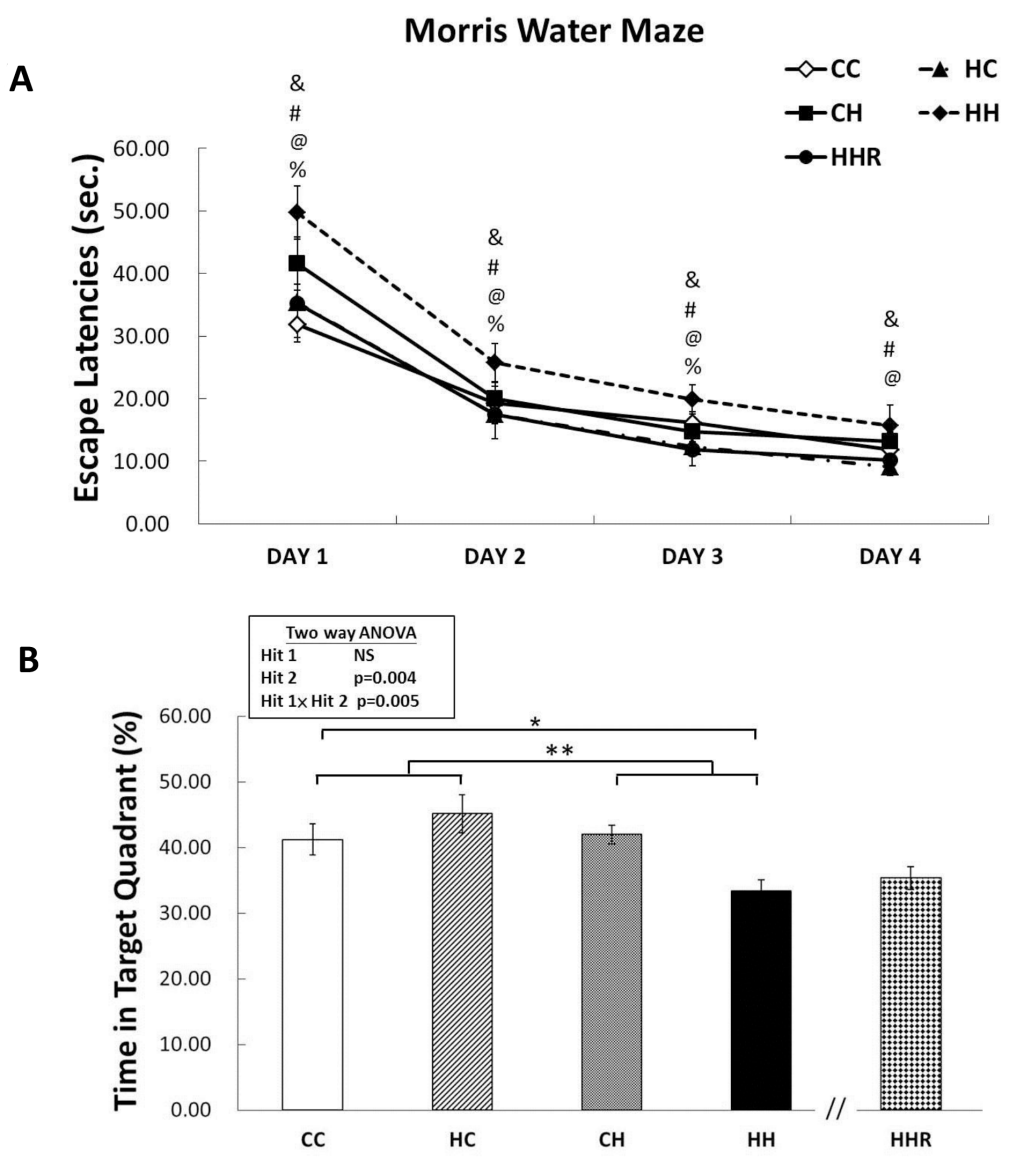
Spatial learning and memory tested using the Morris water maze **(A)** Escape latencies to the platform in the Morris water maze (mean ± SEM). Rats in the HH group swam for a longer period to find the submerged platform on all four acquisition days compared to rats in the CC, HC, and CH groups. HHR group rats took less time to reach the platform compared to rats in the HH group. **(B)** HH rats on average spent the least amount of time in the target quadrant on day 5 among the four experimental groups. However, there was no significant difference in retention between HH and HHR rats. A repeated measures ANOVA was used to assess the differences among groups in acquisition. The therapeutic effect of resveratrol was evaluated using post hoc analysis. ^&^: P<0.05, HH versus CC; ^#^: P<0.05, HH versus HC; ^@^: P<0.05, HH versus CH; ^%^: P<0.05, HH versus HHR; ^*^ P<0.05; ^**^ p<0.01.

Acquisition: The water maze tests revealed that all rats were able to learn how to find the platform and that there was no significant difference in swim velocity between the different treatment groups at any time (P>0.1). Escape latencies improved over time in all four groups as indicated by a significant effect of day of testing on escape latency, indicating that learning occurred (Figure 3). We observed a significant main effect of maternal obesity/high-fat diet on escape latencies [F(1,303)=13.829, P<0.001]. There was also a significant interaction effect between maternal obesity and postnatal high-fat diet [F(1,303)=5.131, P=0.024]. Post hoc analysis showed that rats in the CH group performed better compared to rats in the HH group (P=0.001) in the Morris water maze test. Post hoc analysis also showed that resveratrol treatment significantly decreased escape latencies (P<0.05).

Retention: In the target zone exploration analysis, repeated measures ANOVA showed a significant main effect of postnatal high-fat diet [F(1,83)=8.606, P=0.004], as well as a significant interaction between maternal obesity and a postnatal high-fat diet [F(1,83)=8.198, P=0.005] in target zone exploration. *Post hoc* analysis showed that rats in the CH group performed better in this test compared to rats in the HH group (P=0.003). This result indicates a retention deficit in HH rats. However, Student’s *t*-test analysis showed that treatment with resveratrol failed to restore the time spent in the target quadrant (P=0.411).

Overall the data suggest that the combination of maternal obesity/high-fat diet and exposure to a postnatal high-fat diet results in the significant impairment of acquisition and retention in the Morris water maze test. Furthermore, treatment with resveratrol is able to rescue spatial acquisition deficit in HH group rats.

### Biochemistry parameters of male offspring

The levels of AST, ALT, total cholesterol, and HDL were higher in HH group rats compared to CC group rats. However, plasma adiponectin levels were decreased in HH rats compared to CC rats (Table 2). Student’s *t*-test analysis showed that resveratrol treatment was able to restore total-cholesterol, triglyceride and HDL levels (HH vs. HHR; p<0.05).

**Table 2:** Biochemistry parameter of male offspring at 4 months

	CC N=14	HC N=14	CH N=14	HH N=12	HHR N=13
**AST (U/L)**	112.92±6.56	98.28±6.36	257.7±24.88^&&^	232.29±22.87^&&^	200.21±18.97
**ALT (U/L)**	32.92±1.04	33.67±1.69	153.57±18.72^&&^	113.86±13.91^&&^	110.5±15.57
**Cholesterol (mg/dL)**	47.56±2.19	51.67±2.82^#^	57.48±2.18^&&^	64.07±3.43^#,&&^	50.21±2.43^%^
**Triglyceride (mg/dL)**	83.76±6.09	91.61±10.02	75.26±8.78	101.79±12.72	64.86±4.83
**HDL (mg/dL)**	31.28±2.27	32.72±2.18	39.3±1.83^&&^	42.93±2.4^&&^	32.00±1.89^%^
**Adiponectin (ng/ml)**	1.51±0.06	1.21±0.06^##^	1.2±0.02^&&^	1.13±0.04^##,&&^	1.14±0.04^%%^

Values are means ± SEM; AST, aspartate aminotransferase; ALT, alanine aminotransferase; HDL, high-density lipoprotein; CC, maternal control diet + postnatal rat chow normal fat diet; HC, maternal high-fat diet + postnatal rat chow normal fat diet; CH, maternal control diet + postnatal high-fat diet; HH, maternal high-fat diet + postnatal high-fat diet; HHR, maternal high-fat diet + postnatal high-fat diet treat with resveratrol. ^#, ##^ differs from chow diet treated dams at P< 0.05 or 0.01, respectively, due to a main effect of maternal obesity/high-fat diet treatment. ^&, &&^ differs from chow diet treated dams at P< 0.05, 0.01 or 0.001, respectively, due to a main effect of postnatal high-fat diet treatment. ^%, %%^: HH vs. HHR evaluated by *post hoc* test, P<0.05 or 0.01, respectively.

### Protein expression levels of adiponectin, phospho-ERK1/2 and phospho-PPARγ in dorsal hippocampus

**Figure 4:**
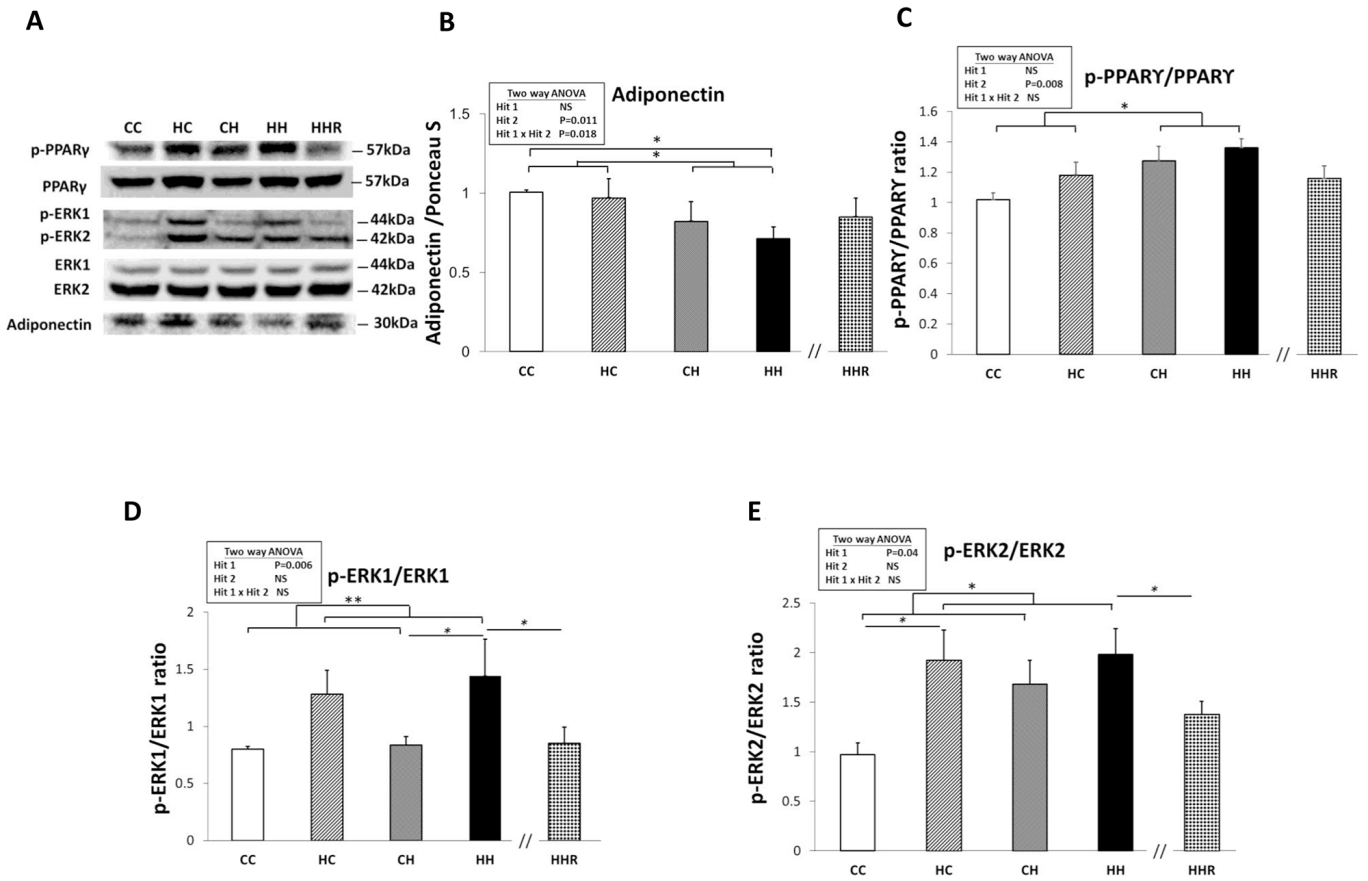
Protein expression levels of adiponectin, phospho-PPARγ, phospho-ERK1, and phospho-ERK2 in rat dorsal hippocampus **(A)** The expression levels of dorsal hippocampal phospho-PPARγ, phospho-ERK1, phospho-ERK2, and adiponectin were detected via western blotting and normalized using Ponceau S staining. Relative abundance of **(B)** adiponectin, **(C)** phospho-PPARγ, **(D)** phospho-ERK1 and **(E)** phospho-ERK2 were quantified. A repeated measures ANOVA was used to assess the differences among groups in the expression levels of these molecules and the therapeutic effect of resveratrol was evaluated using the Student *t*-tests. n=6 for each group. ^*^ P<0.05; ^**^ P<0.01.

Previous studies have associated decreased plasma adiponectin levels with insulin resistance and mild cognitive dysfunction [23–25]. A two-way ANOVA analysis showed a main effect of postnatal high-fat diet treatment [F(1,32)=7.285, P=0.011], but not maternal obesity/high-fat diet [F(1,32)=0.538, P=0.469] on hippocampal adiponectin expression. There was also a significant interaction effect between maternal obesity/high-fat diet and a postnatal high-fat diet [F(1,32)=6.252, P=0.018]. *Post hoc* analysis showed lower hippocampal adiponectin levels in the HH group of rats compared to the CH group of rats (CH vs. HH: p<0.05). This result indicates that exposure to a postnatal high-fat diet results in decreased hippocampal adiponectin. It also demonstrates an additive effect of the 2 treatments (Figure 4A and 4B). However, a Student’s *t*-test analysis showed that resveratrol treatment failed to restore hippocampal adiponectin expression in HHR group rats as compared with HH rats (P>0.05).

Previous studies have shown that PPARγ regulation can be mediated via the ERK1/2 pathway and phosphorylation of PPARγ is involved in insulin sensitivity [26]. A two-way ANOVA showed a significant main effect for postnatal high-fat diet treatment [F(1,32)=8.049, P=0.008], but not for maternal obesity/high-fat diet in phospho-PPARγ expression. There was also no significant interaction effect between maternal obesity/high-fat diet and postnatal high-fat diet. This result indicates that exposure to a postnatal high-fat diet results in increased levels of phospho-PPARγ within the dorsal hippocampus (Figure 4A and 4C). Furthermore, Student’s *t*-test analysis showed that resveratrol treatment did not restore hippocampal phospho-PPARγ levels (HH vs. HHR; P>0.05).

A two-way ANOVA revealed a main effect of maternal obesity/high-fat diet treatment [F(1,32)=8.522, P=0.006], but not postnatal high-fat diet on phospho-ERK1 expression in the hippocampus. There was no significant interaction effect between maternal obesity/high-fat diet and postnatal high-fat diet. This result indicates that maternal obesity/high-fat diet results in a significant increase in hippocampal phospho-ERK1 (Figure 4A and 4D). A Student’s *t*-test showed that resveratrol treatment was able to restore hippocampal phospho-ERK1 levels (HH vs. HHR; P<0.05).

A two-way ANOVA revealed a main effect of maternal obesity/high-fat diet treatment [F(1,24)=4.731, P=0.040], but not postnatal high-fat diet on hippocampal phospho-ERK2 expression. There was no significant interaction effect between maternal obesity/high-fat diet and a postnatal high-fat diet. This result indicates that maternal obesity results in increased hippocampal phospho-ERK2expression (Figure 4A and 4E). Student’s *t*-test showed that resveratrol treatment was able to restore hippocampal phospho-ERK2 levels (HH vs. HHR; P<0.05).

Overall, we observed that maternal obesity/high-fat diet led to an increase in hippocampal phospho-ERK1 and -ERK2 expression. On the other hand, a postnatal high-fat diet results in a decreased expression of hippocampal phospho-PPARγ. Furthermore, animals subjected to both treatments were observed to have the lowest expression levels of hippocampal adiponectin. Additionally, treatment with resveratrol restored the expression levels of phospho-ERK1 and -ERK2 (HH vs. HHR; P<0.05), but not adiponectin or phospho-PPARγ (HH vs. HHR; P>0.05).

### Protein expression levels of phospho-IRS-1, TNF-α, SIRT1 and p66Shc in dorsal hippocampus

**Figure 5:**
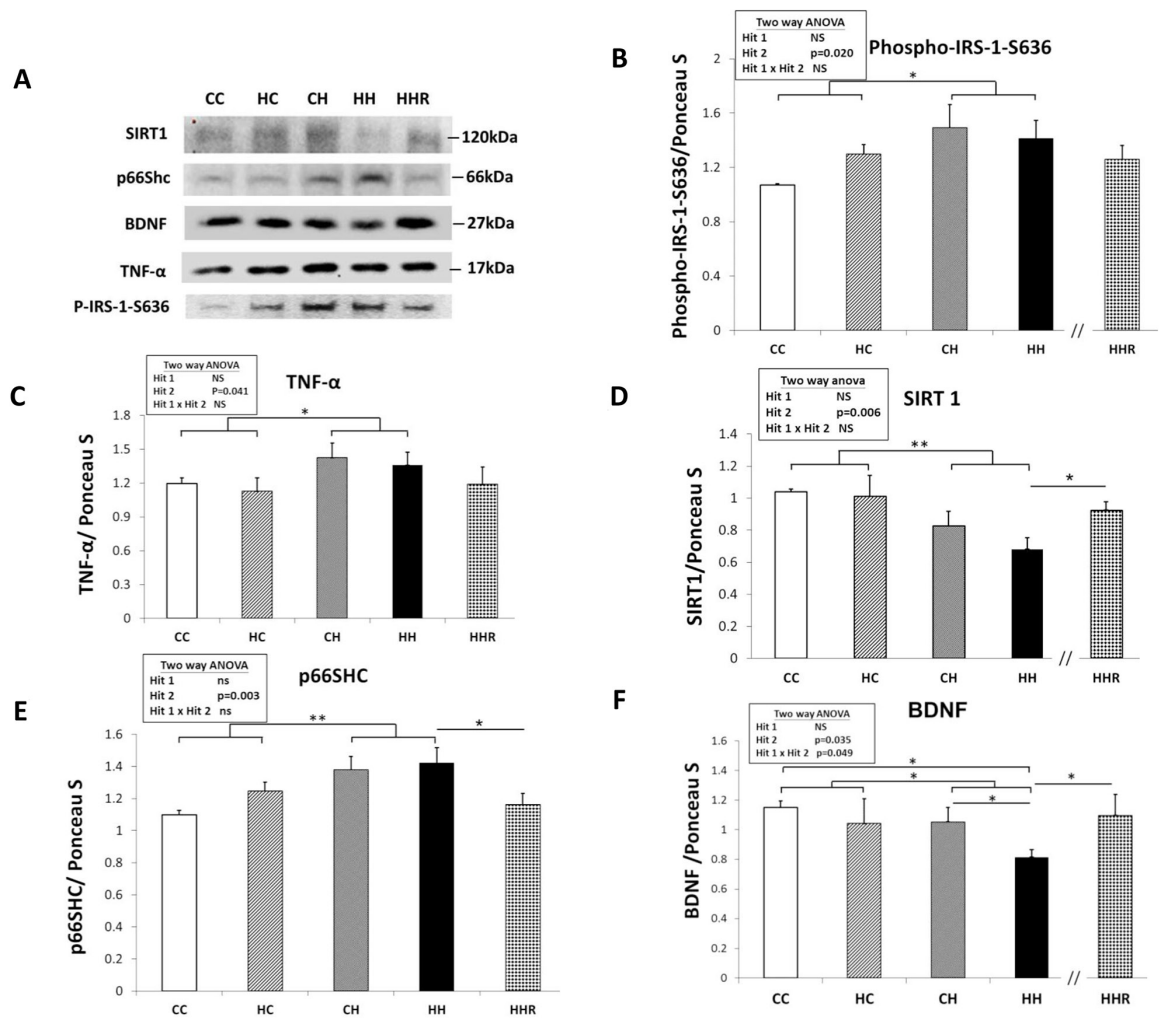
Protein expression levels of phospho-IRS-1-S636, TNF-α, SIRT1, p66Shc, and BDNF in rat dorsal hippocampus **(A)** The expression levels of IRS-1, TNF-α, SIRT1, p66Shc, and BDNF in the dorsal hippocampus were detected via western blotting and normalized using Ponceau S staining. Relative abundance of **(B)** phospho-IRS-1-S636, **(C)** TNF-α, **(D)** SIRT1, **(E)** p66Shc and **(F)** BDNF were quantified. A repeated measures ANOVA was used to assess the differences among groups and the therapeutic effect of resveratrol was evaluated using the Student *t*-tests. n=6 for each group. ^*^ P≤0.05; ^**^ P<0.01.

In order to determine the effect of maternal obesity/high-fat diet and a postnatal high-fat diet on central insulin resistance, we assayed for the level of phospho-insulin receptor subunit-1 (IRS-1) in the dorsal hippocampus [27]. A two-way ANOVA showed a main effect of postnatal high-fat diet treatment [F(1,29)=6.012, P=0.020], indicating an increase in the expression level of phospho-IRS-1, (i.e., decreased IRS-1 downstream signaling), in the HH group compared to the CC group (Figure 5A and 5B). Resveratrol treatment failed to restore the levels of IRS-1 (HH vs. HHR; P>0.05).

To investigate the role of TNF-α in activating the phosphorylation ERK [28], we analyzed the expression level of TNF-α level in the hippocampus (Figure 5A and 5C). A two-way ANOVA showed no interaction effect between maternal obesity/high-fat diet and a postnatal high-fat diet [F(1,32)=0.001, P=0.986]. However, there was a main effect for postnatal high-fat diet treatment [F(1,32)=4.532, P=0.041], indicating that TNF-α expression levels were higher in HH group rats compared to CC group rats. There was, however, no significant main effect of maternal obesity/high-fat diet [F(1,32)=0.369, P=0.548] on hippocampal TNF-α levels. A Student’s *t*-test showed that resveratrol treatment did not restore TNF-α expression in the hippocampus (HH vs. HHR; P=0.317).

A two-way ANOVA showed a main effect of postnatal high-fat diet treatment on expression levels of SIRT1 [F(1,23)=9.030, P=0.006] and of p66Shc [F(1,28)=10.341, P=0.003] in the hippocampus. However, there was no effect of maternal obesity/high-fat diet on the expression levels of both SIRT1 and p66Shc in the hippocampus (all P>0.05). There was also no significant interaction effect between maternal obesity/high-fat diet and postnatal high-fat diet on the expression level of both proteins in the hippocampus (all P>0.05). This result indicates that a postnatal high-fat diet results in decreased hippocampal SIRT1 levels and increased expression of hippocampal p66Shc. A Student’s *t*-test showed that resveratrol treatment restored hippocampal SIRT1 and p66Shc expression levels (HH vs. HHR; P<0.05).

Together, the above data showed that a postnatal high-fat diet led to increased phospho-IRS-1-S636, TNF-α, and p66Shc expression, whereas SIRT1 expression was decreased in the hippocampus. Furthermore, resveratrol treatment restored SIRT1 and p66Shc expression (HH vs. HHR; all P<0.05), but not phospho-IRS-1-S636 and TNF-α expression in the hippocampus (HH vs. HHR; all P>0.05).

### BDNF protein expression levels in dorsal hippocampus

A two-way ANOVA showed a main effect of postnatal high-fat diet treatment [F(1,24)=5.061, P=0.034], but not maternal obesity/high-fat diet on hippocampal BDNF expression (Figure 5A and 5F). This indicates that rats in the HH group had lower levels of BDNF compared to rats in the HC group. We also observed an interaction effect between maternal obesity/high-fat diet and postnatal high-fat diet treatments [F(1,24)=4.290, P=0.049]. *Post hoc* analysis showed that the HH group had the lowest BDNF levels when compared to the CC, HC, and CH groups. Student’s *t*-test analysis showed that resveratrol treatment restored hippocampal BDNF expression in HHR (HH vs. HHR; P<0.05).

## DISCUSSION

We report here that maternal obesity/high-fat diet interacts with a postnatal high-fat diet to induce features of metabolic syndrome, alter biochemical profiles in the dorsal hippocampus, and lead to cognitive deficits. Treatment with resveratrol is able to rescue most of these effects in rats. We have shown that (1) maternal obesity/high-fat diet results in the increased expression of phospho-ERK1 and phospho-ERK2 in the dorsal hippocampus. (2) A postnatal high-fat diet results in increased blood pressure, increased central and peripheral insulin resistance, decreased expression levels of SIRT1, adiponectin, and BDNF, as well as increased expression levels of phospho-PPARγ, phospho-IRS1, TNF-α, and p66Shc in the dorsal hippocampus. (3) The combination of maternal obesity/high-fat diet and a postnatal high-fat diet led to cognitive impairment and a further decrease in adiponectin and BDNF expression levels in the dorsal hippocampus. (4) Resveratrol treatment restored peripheral insulin resistance, elevated blood pressure, altered dorsal hippocampal phospho-ERK1, -ERK2, SIRT1, p66Shc and BDNF expression to levels observed in control animals, Resveratrol administration was also able to partly rescue cognitive deficits.

An increasing number of studies have demonstrated that offspring exposed to maternal obesity/overnutrition during both pregnancy and lactation are susceptible to increase in adiposity and metabolic dysregulation compared to those from control dams when the offspring themselves are challenged with a high-fat diet after weaning [4, 8, 29–32].

Previous reports showed sex-dependent mechanisms are involved in rat offspring induced by either a maternal high-fat diet and rats fed with a postnatal high-fat diet [14, 33, 34]. Underwood *et al.* demonstrated that a high-fat diet caused impairment in hippocampus-dependent memory in a sex-dependent manner in rats [35]. In this study, we focused on the cognition and molecular mechanisms in male offspring only. Further studies should be conducted in parallel in both males and females.

In this study, combined maternal obesity/high-fat diet and a postnatal high-fat diet increased body weights, plasma cholesterol and triglycerides. The combined treatment also resulted in increased blood pressure, and impaired glucose tolerance. However, resveratrol treatment restored the increased body weights, elevated blood pressures and impaired glucose tolerance. Taken together, a combined maternal obesity/high-fat diet and a postnatal high-fat diet results in a metabolic syndrome phenotype, and resveratrol treatment is able to rescue this phenotype.

We observed that maternal obesity/high-fat diet did not result in spatial impairment. This finding was not surprising, given that there have been conflicting results about the effects of maternal obesity on cognitive abilities of offspring. For instance, adult offspring from obese dams have been reported to show normal spatial ability, better spatial performance [32], or impaired spatial performance [36, 37] depending on the publication. Animal studies have also reported that rodents maintained on a high-fat diet show impaired learning and cognitive functions [36, 37]. However, normal spatial function following postnatal high-fat diet has also been reported [38–40]. Intriguingly, our study showed that the combined effect of a maternal obesity/high-fat diet and a postnatal high-fat diet led to spatial impairment, and treatment with resveratrol was able to reverse the spatial acquisition deficit but not the retention deficit.

Insulin sensitivity is known to be regulated via serine/threonine phosphorylation of IRS-1, in which phospho-IRS-1-Ser636 was associated with desensitization of insulin signaling [27]. Furthermore, activation of the insulin receptor cascade is associated with cognitive function [41]. Insulin resistance in hippocampus is implicated in cognition dysfunction encountered in metabolic syndrome [35, 42–45]. In our study, we demonstrate an upregulation of phospho-IRS-1-Ser636 in rats assigned to the CH and HH groups. This indicates that a postnatal high-fat diet results in central insulin resistance. However, resveratrol treatment was unable to rescue this effect.

TNF-α, PPARγ, and ERK are implicated in insulin resistance in metabolic syndrome [45–47]. TNF-α, a proinflammatory cytokine, is increased in excessive insulin, obesity and metabolic syndrome [48, 49]. Our study showed that a postnatal high-fat diet led to increased hippocampal expression of TNF-α. TNF-α can phosphorylate PPARγ in an ERK-dependent manner in adipose tissue [50], as well as PPARγ can regulate BDNF promoter activity and exert the neuroprotective effects in obese insulin resistant rats [46, 51]. We observed that maternal obesity/high-fat diet caused increased hippocampal phospho-ERK1 and -ERK2 expression, whereas resveratrol treatment restored them. Our results are consistent with the study conducted by Hosooka *et al.* that showed that high-fat diet and high glucose incubation increased ERK1/2 phosphorylation as well as phosphorylation of PPARγ [52]. Rats fed a high-fat diet showed decreased adipose tissue adiponectin and increased phosphorylation of ERK and PPARγ. These effects were rescued by administration of green tea polyphenols [53]. PPARγ belongs to the PPAR family of nuclear hormone receptors best known for their role in regulating various genes involved in glucose homeostasis and lipid metabolism [24]. In our study, we observed that a postnatal high-fat diet led to increased phosphorylation of PPARγ in rats assigned to the CH and HH groups, suggesting a diabetic effect [54].

Adiponectin is also implicated in insulin resistance in metabolic syndrome [47, 55]. Adiponectin is an adipokine that is inversely correlated with body mass index and fat mass. It induces lipolysis and improves insulin sensitivity in peripheral tissues [55, 56]. In our study, HC, CH, and HH group rats had lower plasma adiponectin as compared with CC group. In parallel, hippocampal adiponectin was decreased in postnatal high-fat rats and was even lower in the HH group, indicating an additive effect of the dual treatment. However, resveratrol treatment failed to restore decreased plasma or hippocampal adiponectin in rats subjected to both treatments. Previous studies have shown that plasma adiponectin levels are decreased in obesity, insulin resistance and type 2 diabetes [24, 25], Additionally, decreased plasma adiponectin levels have been associated with mild cognitive dysfunction [23]. Hippocampal adiponectin is also known to be involved in cognitive function [39].

Both SIRT1 and p66Shc are implicated in insulin resistance in metabolic syndrome [57, 58]. A postnatal high-fat diet resulted in decreased expression of hippocampal SIRT1; however, resveratrol treatment was able to restore expression as observed in the HHR group. Mice maintained on a high-fat diet are known to exhibit impaired spatial memory associated with reduced hippocampal SIRT1 mRNA expression [59]. In addition, a high-fat diet may cause insulin resistance, lead to impaired hippocampus-dependent spatial memory and dysregulation of hippocampal SIRT1 via an epigenetic mechanism [57, 60]. P66Shc mediates obesity-induced insulin resistance [58]. A previous study in human umbilical vein endothelial cells showed that p66Shc was repressed via the binding of SIRT1 to the p66Shc promoter [61]. We have demonstrated that a postnatal high-fat diet leads to increased hippocampal p66Shc in rats assigned to the CH and HH groups, and resveratrol treatment is able to restore the levels.

BDNF plays a critical role in hippocampal long-term potentiation, which is a long-term enhancement of synaptic efficacy thought to underlie learning and memory [62]. BDNF is also involved in the pathogenesis of obesity, type 2 diabetes mellitus, and metabolic syndrome [44, 63, 64]. Previous studies have demonstrated that the reduction in BDNF resulting from intake of a high-fat diet may impair learning and memory by interfering with hippocampal function [36, 65, 66]. Furthermore, juvenile offspring of obese mothers were reported to have decreased hippocampal BDNF expression and impaired spatial ability; these effects were absent in adult offspring of obese mothers [66]. We observed a lower expression of hippocampal BDNF in rats assigned to the HH group compared to rats assigned to the CC, HC, and HH groups, suggesting, an additive effect of maternal high-fat diet/obesity and a postnatal high-fat diet. Treatment with resveratrol was able to restore the levels of BDNF.

Resveratrol activates SIRT1 and has a variety of biological and pharmacological effects, including cardio-protective, antioxidant, and anti-inflammatory effects [67–69]. It is also known to preventing type 2 diabetes mellitus and metabolic syndrome [70]. Resveratrol is also able to increase nitric oxide bioavailability, as well as facilitate the endothelium-dependent vasodilatation necessary for adequate cerebral perfusion [71]. Resveratrol is also able to attenuate hippocampus insulin resistance and spatial deficit in mice induced by a high-fat diet [49]. In addition, resveratrol increased hippocampal SIRT1 activity and preserved hippocampus-dependent memory in mice placed on a high-fat diet [60]. Resveratrol also confers antidepressant-like effects possibly via activation of hippocampal BDNF in rats [72]. In our study, we observe that resveratrol treatment was able to rescue the effects of a high-fat diet on body weight, elevated blood pressure, impaired blood glucose homeostasis and cognitive deficits. Additionally, resveratrol restored expression of phospho-ERK1/2, SIRT1, p66Shc, and BDNF in the hippocampus of mice assigned to the HHR group.

In conclusion, exposure to maternal and lactational high-fat diet appears to sensitize the offspring to a postnatal high-fat diet and promotes the development of features related to the metabolic syndrome, including cognitive deficits. In this study, we found altered mediators implicated in insulin resistance and cognition, including adiponectin, PPARγ, ERK1/2, p66Shc, SIRT1 and BDNF. Moreover, resveratrol administration is able to reduce the metabolic abnormalities and reverse cognitive deficits in rats exposed to both a maternal and lactational high-fat diet and a postnatal high-fat diet.

## MATERIALS AND METHODS

### Animals and experimental design

This study was carried out in strict accordance with the recommendations of the Guide for the Care and Use of Laboratory Animals of the National Institutes of Health. The protocol was approved by the Institutional Animal Care and Use Committee of the Kaohsiung Chang Gung Memorial Hospital. Virgin Sprague-Dawley (SD) rats (BioLASCO Taiwan Co., Ltd., Taipei, Taiwan) were housed and maintained in a facility accredited by the Association for Assessment and Accreditation of Laboratory Animal Care International. The rats were exposed to a 12 h light/12 h dark cycle. Male SD rats were caged with female rats until mating was confirmed by examining vaginal plug.

**Figure 6:**
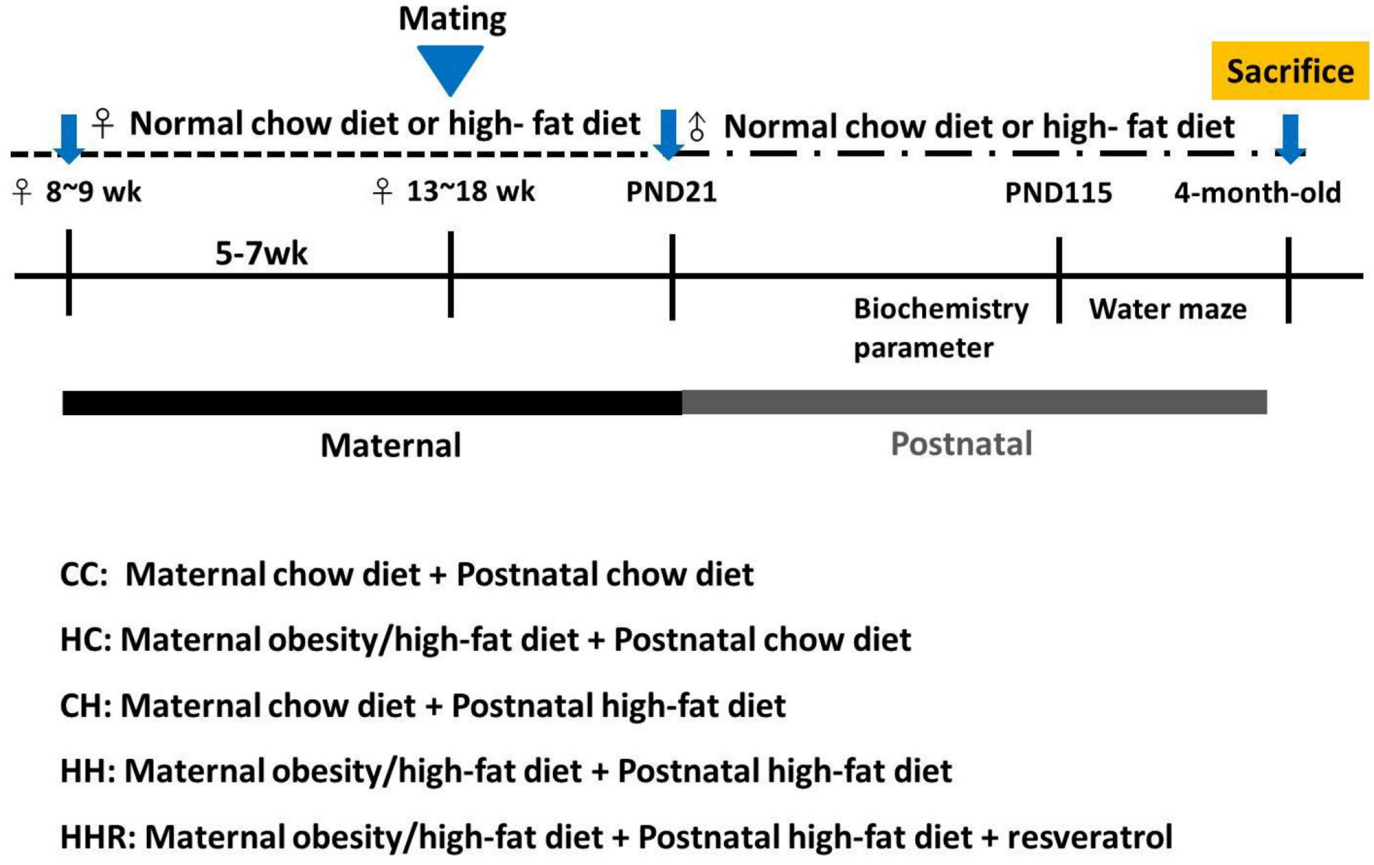
Flowchart of the experimental design 8-9 weeks old female rats were fed with chow diet or high-fat diet for 5 weeks. After mating and delivery of pups, male baby rats were weaned and fed a chow diet or a high-fat diet from PND21 onwards. Five experimental groups (n=12-14 per group) were generated: CC: maternal rat chow diet + postnatal chow diet; HC: maternal obesity/high-fat diet + postnatal chow diet; CH: maternal rat chow diet + postnatal high-fat diet; HH: maternal obesity/high-fat diet + postnatal high-fat diet; HHR: maternal obesity/high-fat diet + postnatal high-fat diet + resveratrol.

Experimental design was as in Figure 6. Female rats were weight-matched and assigned to receive either a normal diet with regular rat chow (ND; Fwusow Taiwan Co., Ltd., Taichung, Taiwan; 52% carbohydrates, 23.5% protein, 4.5% fat, 10% ash, and 8% fiber) or high-fat hypercaloric diet (HF; D12331, Research Diets, Inc., New Brunswick, NJ, USA; 58% fat [hydrogenated coconut oil] plus high sucrose [25% carbohydrate]) *ad libitum* for 5 weeks before mating and during gestation and lactation. After birth, litters were culled to 8 pups, with equal numbers of each sex to standardize the received quantity of milk and maternal pup care. The offspring of both sexes were weaned at 3 weeks of age, and onto either the normal diet or high-fat diet *ad libitum* from weaning to 4 months of age. Five experimental groups (n = 12-14 per group) were generated: control rat chow diet/postnatal rat chow diet (CC), maternal high-fat diet/postnatal rat chow diet (HC), control rat chow diet/postnatal high-fat diet (CH), and maternal high-fat diet/postnatal high-fat diet [73]. In addition, a therapeutic group with resveratrol on maternal high-fat diet/postnatal high-fat diet was raised for comparison (HHR).

### Blood pressure

Blood pressure was measured in conscious rats at 4 months of age by using an indirect tail-cuff method (BP-2000, Visitech Systems, Inc., Apex, NC, USA) after systematically trained. For each rat, 5 measurements were recorded at each time point as we previously described [14, 74]. Three stable consecutive measures were taken and averaged.

### Intraperitoneally injected glucose tolerance test (IPGTT)

After an 8-h fast at postnatal day (PND) ∼110, blood samples were collected at 5 time points: before injection and at 15, 30, 60, and 120 min after the intraperitoneally (i.p.) injection of glucose (2 g/kg body weight). Plasma glucose levels were immediately measured using the enzymatic (hexokinase) method with a glucose assay kit. Serum insulin levels were checked using enzyme-linked immunosorbent assay (Crystal Chem Inc., Downers Grove, IL, USA), as we previously reported [75].

### Morris water maze: spatial memory

The Morris water-maze test was conducted to assess spatial learning and memory in all five groups [76, 77]. Briefly, at PND ∼115, each rat was habituated to the training environment. Between PND ∼116 and 119, the rats were subjected to six trials per day to find the submerged platform. The starting position was changed with each trial. This period was considered as the acquisition phase. At PND ∼120, retention of memory was tested with the platform absent.

### Tissue collection and blood sampling

After lactation, the lactating dams were weighed and euthanized under isoflurane by cervical dislocation (n=7-12 per group). The male offspring were weighed and euthanized at the age of 4 month (n=12-14 per group). Plasma and dorsal hippocampus were collected. Enzyme-linked immunosorbent assay (ELISA) for plasma included triglyceride, liver transaminase, adiponectin, cholesterol, sugar and insulin were according to the manufacturers’ protocols.

### Western blot

Western blot analysis was done as described previously [78]. Total protein extracts from homogenized from cultured cells and liver were lysed in ice-cold RIPA buffer with protease inhibitor cocktail (Roche, Indianapolis, IN, USA). After centrifugation, protein concentrations in supernatants were determined by the DC protein assay kit (Bio-Rad, Hercules, CA, USA). The Western blotting technique was performed to quantify the protein density of adiponectin, ERK1/2, BDNF, PPARγ phosphorylation, PPARγ and adiponectin. Briefly, proteins of the left ventricle were isolated, separated by electrophoresis, transferred to a nitrocellulose membrane and probed with one of the following primary antibodies: ERK1/2, BDNF, PPAR phosphorylation, PPARγ and adiponectin (phosphorylated 1:1000 and total 1:2000). All blots were stained with Ponceau S as an internal standard, as previously described [76, 79]. Bands of interest were visualized using electrochemiluminescence reagents (PerkinElmer, Waltham, MA) and quantified by densitometry (Quantity One Analysis software; Bio-Rad), as the integrated optical density (IOD) after subtraction of background. The IOD was baseline adjusted from Ponceau S-staining to correct any variations in total protein loading and an internal standard [76, 80, 81]. The amount of protein was represented as IOD/Std.

### ELISA

In order to detect the adiponectin concentration, we used the rat Adiponectin/Acrp30 DuoSet ELISA (R&D, DY3100-05) kit to detect the proportional adiponectin concentration from dorsal hippocampus. The adiponectin concentrations were interpolated from standard curve and normalized to total sample mass.

### Statistical analysis

Student’s *t*-test was used to compare the body weight and biochemistry parameters between chow and high-fat diet dams. Results were analyzed using two-way analysis of variance (ANOVA) (maternal diet x postweaning diet), followed by Fisher’s LSD post hoc tests if the interaction was significant. For all variables measured, outliers below the first quartile or above the third quartile were removed from the analysis. If there was an additive effect of maternal obesity and postnatal high-fat diet, then the therapeutic effect of resveratrol was evaluated by Fisher’s LSD *post hoc* or unpaired Student *t*-tests. Results from the Morris water maze testing and IPGTT were subjected to repeated measures ANOVA of two-way designed in maternal chowing (normal chow diet vs. high-fat diet), postweaning diet (normal chow diet vs. high-fat diet) were between-subjects factors. The within-subjects factor was days. The major results of probe tests (e.g. dwell time in platform quadrant versus all other quadrants) were compared using one-way ANOVA followed by Fisher’s LSD *post hoc* tests [82]. All analyses were performed using Statistical Package for the Social Sciences (SPSS) software. Values were expressed as mean ± SEM. Significance was defined as P< 0.05 for all tests.
